# Detecting Glucose Levels in Blood Plasma and Artificial Tear by Au(I) Complex on the Carbopol Polymer: A Microfluidic Paper-Based Method

**DOI:** 10.3390/polym10091001

**Published:** 2018-09-07

**Authors:** Jong-Jheng Luo, Sheng-Wei Pan, Jia-Hui Yang, Tian-Lin Chang, Peng-Yi Lin, Chen-Liang Wu, Wei-Fang Liu, Xin-Ru Huang, Igor O. Koshevoy, Pi-Tai Chou, Mei-Lin Ho

**Affiliations:** 1 Department of Chemistry, Soochow University, No 70, LinShih Rd., Shih-Lin, Taipei 11102, Taiwan; rupert123123123a@gmail.com (J.-J.L.); cindyyang850115@gmail.com (J.-H.Y.); leo011211@gmail.com (T.-L.C.); 06333003@scu.edu.tw (P.-Y.L.); tony807761620047@gmail.com (C.-L.W.); yvonne0123weifang@gmail.com (W.-F.L.); lina19961016@gmail.com (X.-R.H.); 2 Department of Chest Medicine, Taipei Veterans General Hospital, Taipei 11217, Taiwan; 3 School of Medicine, National Yang-Ming University, Taipei 11221, Taiwan; 4 Institute of Public Health, National Yang-Ming University, Taipei 11102, Taiwan; 5 Department of Chemistry, University of Eastern Finland, 80101, Joensuu, Finland; igor.koshevoy@uef.fi; 6 National Taiwan University, Department of Chemistry, Taipei 11102, Taiwan; chop@ntu.edu.tw

**Keywords:** blood plasma glucose, tear glucose, carbopol polymer, diabetes, microfluid device, luminescent

## Abstract

We report on a selective paper-based method and a microfluidic paper-based analytical device (μPAD) for the detection of human plasma glucose and tear glucose using carbopol polymer-encapsulated Au(I) complex (AuC_2_C_6_H_4_OMe)_2_(Ph_2_P(C_6_H_4_)_3_PPh_2_), (**B5**). To the best of our knowledge, this demonstrates for the first time the glucose sensing based on dual emission, i.e., fluorescence and phosphorescence, of a single type molecule on the carbopol polymer. Upon addition of human blood treated with anticoagulants to μPADs, plasma is separated from the blood and flows into the response region of the μPADs to react with carbopol polymer-encapsulated **B5**, in which the ratiometric luminescence is analyzed. The plasma glucose concentration can be quantitively detected at 1.0–50.0 mM on paper, and tear glucose can be detected at 0.1–4.0 mM on μPADs. Owing to the structural design, this device has superior ratiometric changes of dual emission over other Au(I) complexes for signal transduction. The encapsulation of carbopol polymer also offers long-term storage stability. In tear measurement, carbopol polymer is not only used to encapsulate enzyme to remain the enzyme’s activity, but also played as a glue (or media) to connect microfluidic channel and response region. This further improves the sensitivity and limit of detection for glucose. Moreover, this sensor provides a faster response time, a wider range for glucose sensing than reported previously, and no statistical difference of the data from a commercial glucometer, allowing for practical diagnosis of diabetes and healthy individuals.

## 1. Introduction

Direct measurement of blood glucose and non-invasive monitoring of glucose in the human body are necessary for diagnosing many diseases and for bio-engineering [1]. Among the accessible body fluids for a non-invasive methodology tears are considered to be an ideal candidate. The glucose concentration in tears has been confirmed to have a positive correlation with that in blood [2]. Some optical sensors for tear glucose, in which a change in color or luminescence is observed upon detection, have been investigated due to their simplicity, high sensitivity, high selectivity, “naked-eye” detection and practically low cost [3]. For example, Gabriel et al. recently reported the measurement of tear glucose based on a paper-based colorimetric biosensor [4]. In contrast, in the fluorescence method, the luminescence intensity at a single wavelength could be affected and even overwhelmed by the background noise of the sample medium [3]. By comparison, a ratiometric sensing system [3] or dual emission system [5,6], e.g., fluorescence and phosphorescence or dual phosphorescence, could surmount this drawback because the ratio of emission intensity at two different wavelengths can internally provide a built-in correction for possible influencing factors [3].

Among the dual emission detecting systems reported to date, several examples rely on mixing multiple emitters [7]. Unlike a single molecule emitter, however, multi-component emitters generally are subject to phase separation, extra components, instability and complex preparation procedures [6]. Therefore, the exploration of a single component exhibiting a dual emission character for signal transduction is always important and attractive.

**Figure 1:**
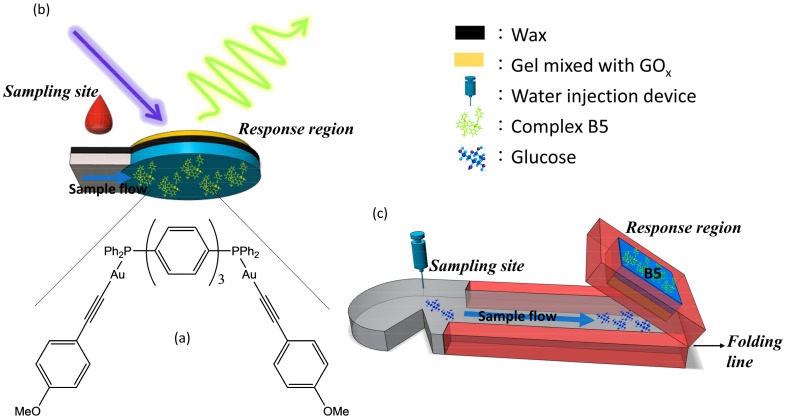
(**a**) Structure of **B5**, (**b**) a schematic illustration of luminescent and microfluidic paper-based analytical device (μPAD) for blood glucose; blue disk is the circular region of enzyme loading in the cellulose paper, and (**c**) a Schirmer strip for tear glucose detection.

Recently, we have developed a series of dinuclear gold(I) alkynyl-diphosphines (X-C_6_H_4_C_2_-Au)PPh_2_-spacer-PPh_2_(Au-C_2_C_6_H_4_-X) (spacer = π-systems, X = OCF_3_, H, and OMe), in which dual emission, i.e., fluorescence and phosphorescence, can be modulated not only via molecular geometry but also via modification of the ancillary groups with different donor-acceptor characteristics [8]. Among these complexes, compound (AuC_2_C_6_H_4_OMe)_2_(Ph_2_P(C_6_H_4_)_3_PPh_2_) (**B5**, Figure 1a) exhibits the best intensity ratio changes for phosphorescence versus fluorescence in sensing the molecular oxygen [8]. The lowest lying electronic transition of **B5** predominantly has an intraphosphine π–π* nature, which conventionally serves as the origin of fluorescence emission. The electron-donating group (X = MeO) affects the energy level of the metal Au(I) d_π_ orbital, leading to a certain mixing of π–π* and MLCT excited states, hence causing an increase in the rate of intersystem crossing *k_isc_* *S*_1_ → *T_m_* (*m* ≥ 1) and thus phosphorescence emission. Furthermore, upon excitation of **B5** at higher lying electronic states, the RT phosphorescence can be significantly enhanced due to the increased population in the *T*_1_ state via faster rate of *S_i_* → *T_j_* (*I* > 1, *j* ≥ 1) intersystem crossing (cf. *S*_1_ → *T_j_* (*j* ≥ 1) [9]. Despite these intriguing and unique photophysical properties, however, exploiting **B5** and its numerous congeners in solid state applications such as sensing are scarce [9,10].

In yet another approach, μPADs have received considerable attention in the development of point-of-care tests for their low material and fabrication costs, portability, minimal equipment requirements and global affordability [4,11]. With the development of μPADs, the luminescence detection technique has been applied to ultra-sensitive detection of bio-analytes [12,13]. The results elaborated above inspired us to develop a facile methodology to prepare complex **B5** on a μPAD, which can be employed selectively to measure plasma glucose levels in human blood and tear glucose. The detection principle of plasma glucose has been well found in ref [9] and works on the increase of emission intensity of phosphorescence due to the consumption in oxygen concentration via oxidation reaction of glucose by glucose oxidase (GOx); also see Equation (1). In this study we also developed a wax-printed device for measuring tear glucose. The wax could be printed directly on Schirmer strips for tear tests. We also made a fair comparison between current device and those of a commercial glucose meters in terms of accuracy over a range of glucose concentrations.

(1) $$\;\;\beta -D-\mathrm{glucose}+O_{2}+H_{2}O\;\overset{\mathrm{GOx}}{\to }D-\mathrm{glucose}\;\mathrm{acid}+H_{2}O_{2}\;$$

## 2. Experimental

### 2.1. Chemicals and Materials

D−(+) glucose, Glucose oxidase (GOx), D-fructose (99%), maltose monohydrate (99%), L-lysine (≧99.8%), L-cysteine (≧99.8%), L-glutathione (≧98%), L-phenylalanine (≧98.5%), galactose (99%), lactose (99%), ascorbic acid (99%), citric acid (99%), uric acid (99%), albumin from human serum (HSA, ≧96%), glycine (≧99.7%), L-tryptophan (≧98%), IgG (≧95%), potassium chloride (≧99.5%), and magnesium dichloride (≧99.8%) were purchased from Sigma Aldrich, Inc. (Saint Louis, MO, USA). Polyacrylic acid (Carbopol 940) was provided by Lubrizol Advanced Materials (Wickliffe, OH, USA). Sodium dihydrogen phosphate and disodium hydrogen phosphate were obtained from Merck (Darmstadt, Germany). Sodium chloride (99.8%), calcium chloride dehydrate (≧99%), and sodium bicarbonate (≧99.7%) were purchased from J.T. Baker (Center Valley, PA, USA). Schirmer strips for tear tests were purchased from Eagle Vision, Inc (Memphis, TN, USA).

### 2.2. The Synthesis of (AuC_2_C_6_H_4_OMe)(Ph_2_(C_6_H_4_)_3_PPh_2_) (Complex ***B5***)

The synthesis was carried out as described previously [8]. In brief, (AuC_2_C_6_H_4_OMe)_n_ prepared analogously to (AuC_2_Ph)_n_ [14]. (AuC_2_C_6_H_4_OMe)_n_ (78 mg, 0.238 mmol) was suspended in CH_2_Cl_2_ (VWR Chemicals, Radnor, PA, USA) (10 cm^3^) and Ph_2_(C_6_H_4_)_3_PPh_2_ [15] (73 mg, 0.122 mmol) was added. The suspension turned into a pale yellowish solution within minutes. The reaction mixture was then stirred for 30 min in the absence of light, treated with activated charcoal (Merck, Darmstadt, Germany), passed through a layer of Al_2_O_3_ (Fluka, Switzerland) (neutral) and evaporated. The resulting amorphous solid was dissolved in CH_2_Cl_2_ (3 cm^3^) and diluted with toluene (VWR Chemicals, Radnor, PA, USA) (3 cm^3^), and an excess of diethyl ether (VWR Chemicals, Radnor, PA, USA) was added slowly to cause the precipitation of a nearly colorless microcrystalline solid. ^1^H NMR (CDCl_3_; 298 K, δ): 7.71 (s, 4H, C_6_H_4_–C_6_H_4_–C_6_H_4_), 7.75–7.58 (m, 16H), 7.56–7.48 (m, 12H), 7.47 (d, *J*_HH_ 8.8 Hz, 4H, *meta*-H C_6_H_4_OMe), 6.81 (d, *J*_HH_ 8.8 Hz, 4H, *ortho*-H–C_6_H_4_OMe), 3.80 (s, 6H, OMe). ^31^P{^1^H} NMR (CDCl_3_; 298 K, δ): 42.4 (s).

### 2.3. Standard Glucose Detection

First, 10^−3^ M complex **B5** solution in dichloromethane was added to a micro-dish (EU Optical Wide area 8-Cap Strip, Taipei, Taiwan) (diameter of 0.5 cm) deposited on a cellulose paper (Qualitative Filter Paper, München, Germany). Phosphate buffer was prepared from 175 mM monosodium phosphate monohydrate and 175 mM disodium hydrogen phosphate (Merck, Darmstadt, Germany). Glucose oxidase (GOx) in 1 mL buffer solution was mixed with carbopol polymer (High Density Gel-Based Agent, Taipei, Taiwan), and the resulting mixture was then deposited with **B5** complex, forming the gel-encapsulated **B5**. Second, 10 µL of glucose solution in buffer was added to the reaction mixture, and the luminescence spectrum was recorded using a 325 nm He-Cd laser (UniKLasers, Edinburgh, UK) as the excitation wavelength with a cut-off filter of 333 nm. The luminescence intensities before and after the addition of glucose were recorded and the emission area was integrated. The luminescence spectrum contained a higher energy fluorescence band (the F band) and a lower energy phosphorescence band (the P band). The integration wavelength ranges from 300 nm to 1000 nm. The change in luminescence intensity was calculated as the ratio of with/without glucose.

### 2.4. Fabrication of μPADs

Glucose sensing μPADs were prepared with reference to Kang et al. [16] and modified as follows. 5 µL of Complex **B5** (1.6 × 10^−3^ M) in dichloromethane was dropped onto the response region of the μPADs and Schirmer strips (Figure 1b,c). The wax-patterned μPADs were then fabricated by wax printing (ColorQube 8570, Xerox, Norwalk, CT, USA) and then heated at 60 °C for 5 s to form a wax hydrophobic barrier.

### 2.5. Determination of Blood Glucose

Two independent methods were compared for the blood glucose determination, including the gel-encapsulated **B5** sensing system and a glucometer from Roche (Accu-Chek Active, Dubai, UAE). The protocol and collection of blood were approved by the Taipei Veterans General Hospital Institutional Review Board (Taiwan; IRB registration number 2017-12-002CC). Blood samples were treated with anticoagulants (EDTA (Ethylenediaminetetraacetic acid, Riedel-de-Haën, Mexico, Germany) 1.8 g/L and NaF (sodium fluoride, Ward Hill, MA, USA), and the resulting solutions were added to the sampling sites of μPADs. The signals were measured through luminescence detection in the response region by a bifurcated optical fiber system (QBIF600-UV-VIS, Ocean optics, Largo, Seelze, FL, USA). Specific concentrations of glucose were added to prepare the spiked samples. 

The glucometer is based on dye-mediated pyrroloquinoline quinone-dependent glucose dehydrogenase reaction. When a blood sample is applied to the test strip, chemical reaction takes places, causing changes of the dye color. The color change correlated with the concentration of the blood glucose. This method is linear within the range of 0.6–33.3 mM, and the detection limit is 0.6 mM.

### 2.6. Statistical Analysis

Continuous data are presented as mean ± SD. To evaluate the statistical evidence for linear relationships between pairs of continuous variables, Pearson’s correlation coefficient, *r*, was calculated and a *p* value of <0.05 was considered significant. A paired *t* test was used to compare the means of two measurements. A linear regression model was also used to assess the associations between continuous variables. All data were analyzed in SPSS (Version 20.0, Chicago, IL, USA).

### 2.7. Determination of Tear Glucose

The backs of the Schirmer strips were covered with adhesive tape to prevent sample leakage. The carbopol polymer mixed with GOx was deposited in the response region. After the paper was folded along a folding line (Figure 1c), human tears were collected from the eye with the Schirmer strip. To study the glucose-responsive properties, glucose in the range of 0.1 to 5.0 mM was dissolved in a simulated tear fluid [17]. 5 µL of simulated tear fluid contained 6.78 g/L NaCl, 2.18 g/L NaHCO_3_, 1.38 g/L KCl, 0.084 g/L CaCl_2_·2H_2_O, and 3.94 g/L albumin at pH 7.4 and was injected onto the sampling site [17]. 3 µL of water was also injected onto the sampling site. Finally, the Schirmer strip was unfolded, the luminescence of the response region was measured, and the glucose concentration was calculated.

## 3. Results and Discussion

### 3.1. Synthesis and Structural Description of ***B5***

Complex **B5** was obtained as reported earlier [8] following a general method of depolymerization of gold(I) alkynyl precursor with a stoichiometric amount of the terphenyl-based phosphine ligand. The resulting compound was isolated as a nearly colorless microcrystalline solid; its NMR spectroscopic data were consistent with the symmetric dinuclear structure depicted in Figure 1.

### 3.2. Optimization of the Sensing Method

**Figure 2:**
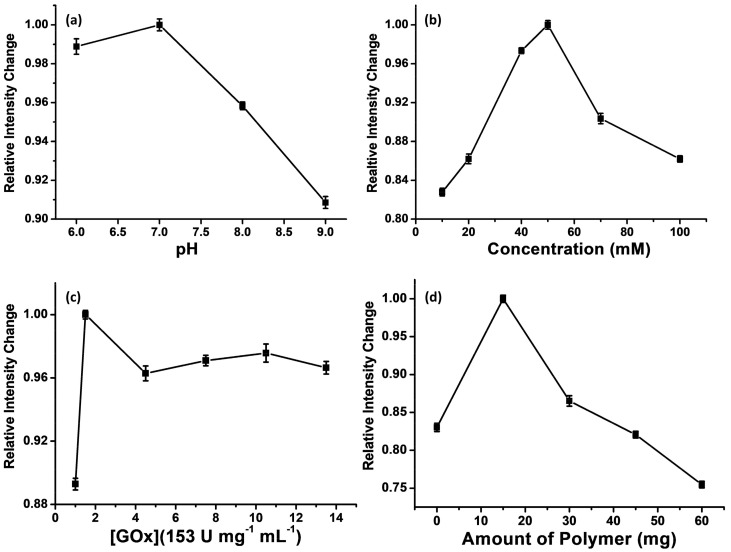
Effects of pH value and the concentration of the buffer (**a**,**b**), concentration of the enzyme, and GOx (EC 1.1.3.4 from Aspergillus niger) with a specific activity of 153 U mg^−1^ of lyophilized solid (**c**), and amount of carbopol polymer (**d**), on the relative intensity change of the paper-based biosensor upon exposure to 20 mM glucose. Data were obtained from the average values of three replicated measurements (N = 3).

In the present gel-encapsulated **B5** sensing system, the change in luminescence intensity is related to the activity of the enzyme. Therefore, the whole system luminescence intensity was measured while pH was varied from 6.0 to 9.0; the buffer concentration, from 10 to 100 mM; and the enzyme concentration, from 1.0 to 14 153U mg^−1^ mL^−1^ (Figure 2). At the same time, the carbopol polymer was for the first time used in the glucose sensing system. Carbopol polymer is a crosslinked polyacrylate that has been used as a rheology modifier in a wide range of personal care and pharmaceutical applications [18]. It has good biocompatibility with many active ingredients and low toxicity. The influence of the amount of carbopol polymer on the change in luminescence intensity was studied. As a result, pH 7.0 and 50 mM of the buffer, 1.5 153U mg^−1^ mL^−1^ of the GOx, and 15 mg of the carbopol polymer were chosen as the optimum conditions for further studies. It is noted that the sensing system using carbopol polymer has a long storage stability (*vide infra*).

### 3.3. Optical Properties of Sensor

**Figure 3:**
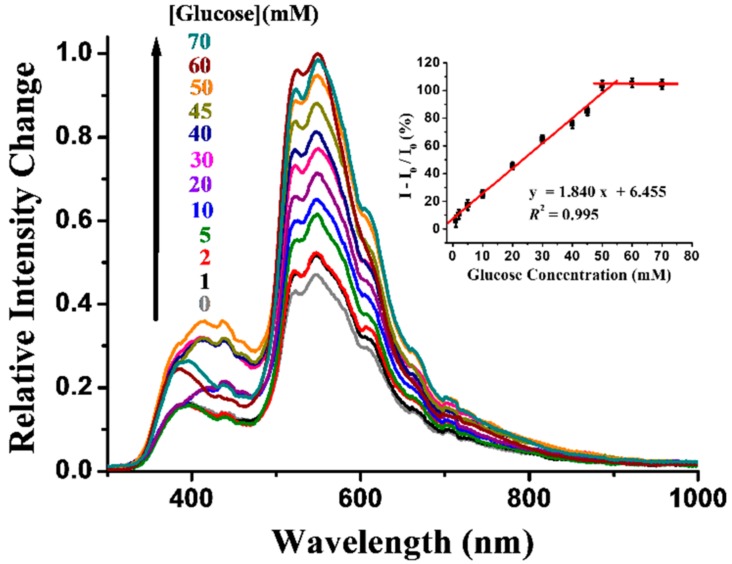
Emission spectra of the gel-encapsulated **B5** sensing system under different concentrations of glucose. Inset: Percentage of emission change as a function of glucose concentration calibration curve obtained for biosensor (N = 3).

Figure 3 shows the emission spectra of the gel-encapsulated **B5** upon the addition of different concentrations of glucose. The emission spectrum of the gel-encapsulated **B5** exhibited a dual emission profile having both a fluorescence band (λ_em_ = 420 nm, the F band) and a phosphorescence band (λ_em_ = 550 nm, the P band), which were due to ^1^π–π* and ^3^π–π* mixed with some MLCT excited states, respectively [8]. With the addition of glucose, the emission intensity of the P band increased gradually due to the consumption of oxygen.

The kinetic behavior and detection range of the gel-encapsulated **B5** (Figure S1 in the supporting information and inset of Figure 3) were simultaneously characterized under the optimum conditions. A comparison to recent reports for glucose detection is provided in Table 1. The gel-encapsulated **B5** system has a faster response time than those in previous reports [9,19,20,21,22]. In addition, this luminescence turn-on type sensing system for glucose is more sensitive than the quenching sensing method [1]. The linear calibration range is wider and is more suitable for monitoring of diabetes in patients.

The inset of Figure 3 shows the calibration curve for glucose detection. The linear response range and the detection limit of this sensing system were subsequently investigated. The percentage of emission intensity change was evaluated by (I − I_0_/I_0_), where I is the integrated area of the F and P bands after glucose addition at time to reach saturation and I_0_ is the original integrated area of the F and P bands without glucose. There was a good linear relationship between the relative luminescence intensity change and the concentration of glucose in the range of 1 to 50 mM. The theoretical limit of detection (LOD) was calculated to be 0.9 mM at a signal/noise ratio of 3.

### 3.4. Interference Study

For glucose detection, we further evaluated the selectivity of our device with various substances existing in human blood, all at concentrations >10-fold higher than typical normal levels in human blood. Furthermore, all substances (mixture) were placed in 20 mM glucose. As shown in Figure S2, the results demonstrated that the changes in emission intensity due to the addition of these substances in blood were negligible. After the addition of the potential interference mixture, another 20 mM of glucose was added to the mixture and the intensity significantly increased, suggesting that the proposed system is highly selective for glucose.

### 3.5. Storage Stability

The stability behavior of current system was studied by storage test at 4 °C and at ambient temperature. During testing, the sensor was maintained at a storage temperature of 4 °C to maintain the enzyme’s activity and operated at ambient temperature. Experiments were performed for at least four months (Figure S3). When stored for 120 days, our sensors exhibited <1.57% loss in the reaction rate, suggesting that the long-term stability of our sensor system is fairly good. The long storage stability may be attributed to the stable gel-encapsulated system, which further maintains the enzyme’s activity.

### 3.6. Determination of Human Plasma Glucose

From the experimental data, the sensor based on the gel-encapsulated **B5** showed a wide response range, fast response time, good reproducibility and good selectivity for the determination of glucose in laboratory samples. To confirm that the method could be used in practice, the sensor was used to detect glucose in human blood. Blood samples were collected from 2 healthy individuals (two 22-year-old men) and 2 patients with type 2 diabetes mellitus (one 80-year-old man and one 49-year-old man), respectively. Blood samples were drawn into collection tubes with anticoagulants, after which the resulting solutions were added to μPADs and measured in the response regions. All the data were rechecked in triplicate to obtain mean values for analysis. Since the blood glucose level is the first line of data on the health information of a patient at hospitals and homes, the accuracy of the present method should be comparable to that of glucometers. Hence, the glucose concentrations were measured simultaneously with a glucometer.

As shown in Table 2, the group 1 samples, i.e., blood from one healthy individual aged 22 years old with glucose added, were tested first. Here, the concentrations of blood glucose were assessed using the standard addition method, and the results are presented (Group 1, samples 1–4). The measured mean plasma glucose levels by the present proposed method were within the range of 8.59 to 20.95 mM. The accuracy of the present method is in accordance with the recommended criteria of values <5.55 mM (100 mg/dL) to ±0.555 mM (10 mg/dL) and values >5.55 to ±1.11 mM of the American Diabetes Association and the International Standardization Organization [23]. The R.S.D. values ranged from 0.58–4.44%, indicating that the proposed method has good accuracy and reproducibility in the determination of glucose in human blood. The mean blood glucose levels in group 1 samples obtained by glucometer are also shown in Table 2. The correlation between glucose values measured using the present method and the glucometer was significant, with a Pearson correlation of *r* = 0.999 (*p* = 0.001) (Table 2). There was no statistical difference between the means of the paired measurements (paired *t* test, *p* = 0.976). 

**Figure 4:**
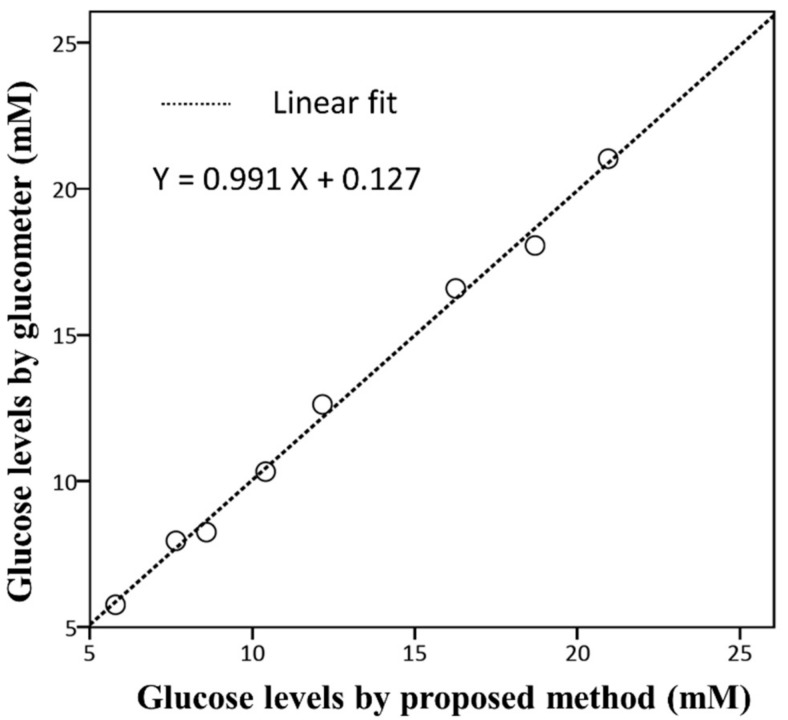
The fitted linear regression line of glucose levels detected by the proposed method versus the reference glucometer (8 blood samples from group 1 and group 2).

Furthermore, the blood samples from 2 healthy individuals and those from 2 diabetes patients were analysed (Group 2, samples 5–8). The mean plasma glucose values measured by the present method and the glucometer were in the ranges of 5.79 to 18.71 mM and 5.76 to 18.06 mM, respectively. The R.S.D. values were less than 5%. The blood glucose values estimated using μPADs and using the glucometer remained strongly correlated in a linear relationship (Pearson’ correlation *r* = 0.998, *p* = 0.002) (Table 2). The means of the paired measurements were not statistically different (paired *t* test, *p* = 0.929). Overall, the correlation between the 8 pairs of glucose values measured by the two methods was significant, with a Pearson correlation of *r* = 0.998 (*p* < 0.001). As shown in Figure 4, there was a fitted regression line (equation Y = 0.991X + 0.127) to the paired data of the 8 samples. In linear regression analysis, the glucose levels detected by the proposed μPADs and by glucometer were significantly associated (*p* < 0.001).

### 3.7. μPADs for Tear Glucose

**Figure 5:**
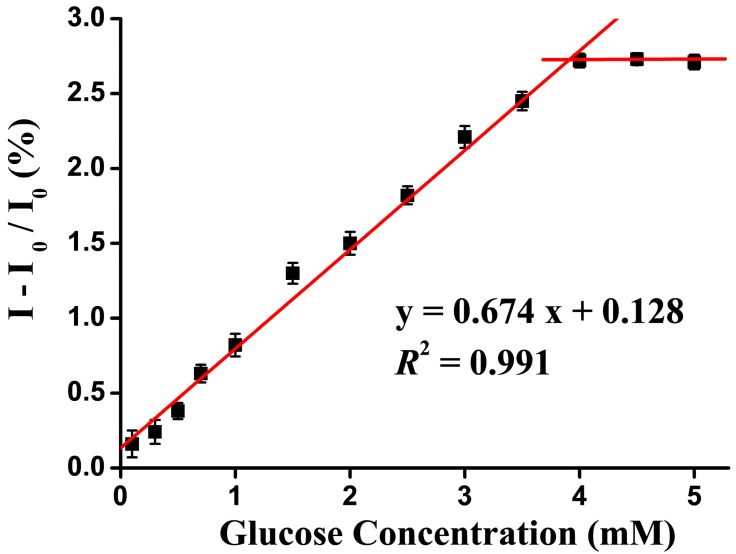
Percentage of emission change as a function of tear glucose concentration calibration curve. (N = 3).

Finally, the gel-capped **B5** was applied to the analysis of tear glucose. The Schirmer strip was divided into two regions: the sampling site (the north position) and the response region (Figure 1c). Wax are located far away from the north position, so that no contact with the eye occurs and thus no possibility of infection. The response region contained gel-encapsulated **B5** for measuring the tear glucose concentration. The microfluidic channel of the μPAD was used for collecting the same amount of human tears [16]. The procedures of folding the strip and injecting a trace amount of water on the strip were used to pre-concentrate the glucose and hence improve the sensitivity and limit of detection for glucose [16]. The carbopol polymer mixed with GOx was designed as an efficient glue to connect the sampling site with the response region (the region of gel mixed with GOx in Figure 1) and provide a biocompatible medium for glucose with **B5**. For actual tear glucose monitoring in the future, the collection times of human tears from both normal and dry eyes should be taken into consideration. Therefore, the luminescence intensity changes in the response region in direct proportion to 0.1–4.0 mM glucose with a collection time of 3 mins are shown in Figure 5. The linear calibration curve for tear glucose was obtained in the concentration range of 0 to 4.0 mM (y = 0.674 [glucose] + 0.128). The limit of detection was 0.08 mM. Known concentration of glucose (3 mM) was spiked into simulated tear by a standard addition method and the response was measured. As can be seen in Table 2, the R.S.D value <5% indicated that the proposed biosensor can also be effectively used for tear glucose analysis. Also, the dynamic range of glucose is presented in Figure 5.

## 4. Conclusions

In summary, polymer gel-encapsulated **B5** on paper and μPADs have been prepared for the measurement of human plasma glucose and tear glucose. Under the optimal conditions for glucose concentration measurements, the luminescence intensity exhibited linear dependence in the range of 1 to 50 mM, with a detection limit of 0.9 mM. The fast response time of the present method is suitable for monitoring glucose levels in emergency cases. The long-term storage stability of the sensor was tested for at least four months. The polymer gel-encapsulated **B5** sensing system and glucometer showed good correlation and no significant difference in determining blood plasma glucose concentrations. Moreover, this sensing system fulfils the accuracy requirements of the American Diabetes Association and the International Standardization Organization. Finally, the sensor provides an efficient way for measuring tear glucose. The configuration of μPADs with gel-encapsulated **B5** allows the Schirmer strip to be used for tear collection, glucose preconcentration, and biocompatible/closer connection, improving the sensitivity and limit of detection of glucose.

## Figures and Tables

**Table 1:** Comparison of the analytical performance of proposed glucose detection method with those of some luminescence glucose biosensors.

Compounds	Linear Range (mM)	LOD (mM)	Response Time (s)	Ref.
**Gel-encapsulated B5**	1.0–50.0 (plasma)	0.9	0.3	Our work
	0.1–2.0 (tear)	0.08	0.3	
**Ag3 on paper ^[a]^**	1.0–35.0	0.09	10	Our previous work [9]
**Type 304 stainless steel electrode-based luminol ^[b]^**	10^−4^–1.0	7.6 × 10^−5^	N.A. ^[c]^	[19]
**GOQD/gold nanoparticles/APBA ^[d]^**	2.5 × 10^−3^–7.5 × 10^−2^	6.5 × 10^−4^	2400	[20]
**AIS QDs ^[e]^**	1.0 × 10^−2^–1.0 ^[f]^	9.0 ×10^−4^	1200	[1]
**C-dots/AgNPs**	2.0 × 10^−3^–0.1	1.39 × 10^−3^	300	[21]
**Ethanolamine-polyborate complexes**	0–8.0	N.A.	3000	[22]

^[a]^ **Ag3** = [Ag(P^3^)CNAg(P^3^)][B(C_6_H_3_(CF_3_)_2_)_4_] (P^3^ = PPh_2_C_6_H_4_−PPh−C_6_H_4_PPh_2_ [bis(o-diphenylphosphinophenyl)phenylphosphine]). ^[b]^ Type 304 stainless steel electrode-based luminol is an electrogenerated chemiluminescence system. ^[c]^ N.A. = Not available. ^[d]^ GOQD/gold nanoparticles/APBA: Graphene oxide quantum dots/gold nanoparticles/3-aminobenzeneboronic acid fluorometry system. ^[e]^ AIS QDs: AgInS2 quantum dots. ^[f]^ AIS QDs showed two linear ranges for glucose detection.

**Table 2:** Detection of glucose in human blood, artificial tear, and the association between various glucose values of the gel-encapsulated **B5** and glucometer.

Sample Number	Glucose Added (mM)	Mean Glucose Levels ^[a]^ (mM)		R.S.D ^[b]^ (%)	Correlation *r*	*p*-value
		Glucometer ^[a]^	Proposed Method ^[a]^			
**Group 1**					0.999	0.001
1	3	8.250 ± 0.06	8.588 ± 0.05	0.58		
2	5	10.32 ± 0.24	10.41 ± 0.36	3.46		
3	10	16.59 ± 0.08	16.26 ± 0.09	0.55		
4	20	21.03 ± 0.12	20.95 ± 0.93	4.44		
**Group 2**					0.998	0.002
5	-	5.764 ± 0.03	5.793 ± 0.19	3.28		
6	-	7.953 ± 0.07	7.650 ± 0.12	1.57		
7	-	12.62 ± 0.25	12.16 ± 0.09	0.74		
8	-	18.06 ± 0.27	18.71 ± 0.51	2.73		
**In artificial tear**		3.088 ± 0.06	3.186 ± 0.11	4.30		

^[a]^ Numbers = 3. ^[b]^ R.S.D.: relative standard deviation of the proposed method.

## Supplementary Materials

The following are available online at http://www.mdpi.com/2073-4360/10/9/1001/s1, Figure S1: The kinetic behavior of the glucose sensing system, Figure S2: Selectivity analysis of the gel-encapsulated **B5** for detection of glucose. Figure S3: The storage stability of the gel-encapsulated B5 for detection of 20 mM glucose.
