# Supplementary material for: Detecting Glucose Levels in Blood Plasma and Artificial Tear by Au(I) Complex on the Carbopol Polymer: A Microfluidic Paper-Based Method
Source: Polymers (Basel). 2018 Sep 7;10(9):1001. doi: 10.3390/polym10091001 (PMC6404068; doi:10.3390/polym10091001)
Supplement: Supplementary file 1 [file polymers-10-01001-s001.pdf]

Supplementary Materials:

Detecting glucose levels in blood plasma and artificial tear by Au(I) complex on the carbopol polymer: a microfluidic paper-based method

Jong-Jheng Luo [1], Sheng-Wei Pan [2,3,4,*] Jia-Hui Yang [1], Tian-Lin Chang [1], Peng-Yi Lin [1], Chen-Liang Wu [1], Wei-Fang Liu [1], Xin-Ru Huang [1], Igor O. Koshevoy [5], Pi-Tai Chou [6] and Mei-Lin Ho [1,*]

[1] Department of Chemistry, Soochow University, No 70, LinShih Rd., Shih-Lin, Taipei 11102, Taiwan; rupert123123123a@gmail.com (J.-J.L.); cindyyang850115@gmail.com (J.-H.Y.); leo011211@gmail.com (T.-L.C.); 06333003@scu.edu.tw (P.-Y.L.); tony807761620047@gmail.com (C.-L.W.); yvonne0123weifang@gmail.com (W.-F.L.); lina19961016@gmail.com (X.-R.H.)
[2] Department of Chest Medicine, Taipei Veterans General Hospital, Taipei 11102, Taiwan
[3] School of Medicine, National Yang-Ming University, Taipei 11102, Taiwan
[4] Institute of Public Health, National Yang-Ming University, Taipei 11102, Taiwan
[5] University of Eastern Finland, Department of Chemistry, 80101, Joensuu, Finland; igor.koshevoy@uef.fi
[6] National Taiwan University, Department of Chemistry, Taipei 11102, Taiwan; chop@ntu.edu.tw
* Correspondence: swpan25@gmail.com (S.-W.P.); meilin_ho@scu.edu.tw (M.-L.H.); Tel.: +886-2-28819471 (ext 6827) (M.-L.H.)

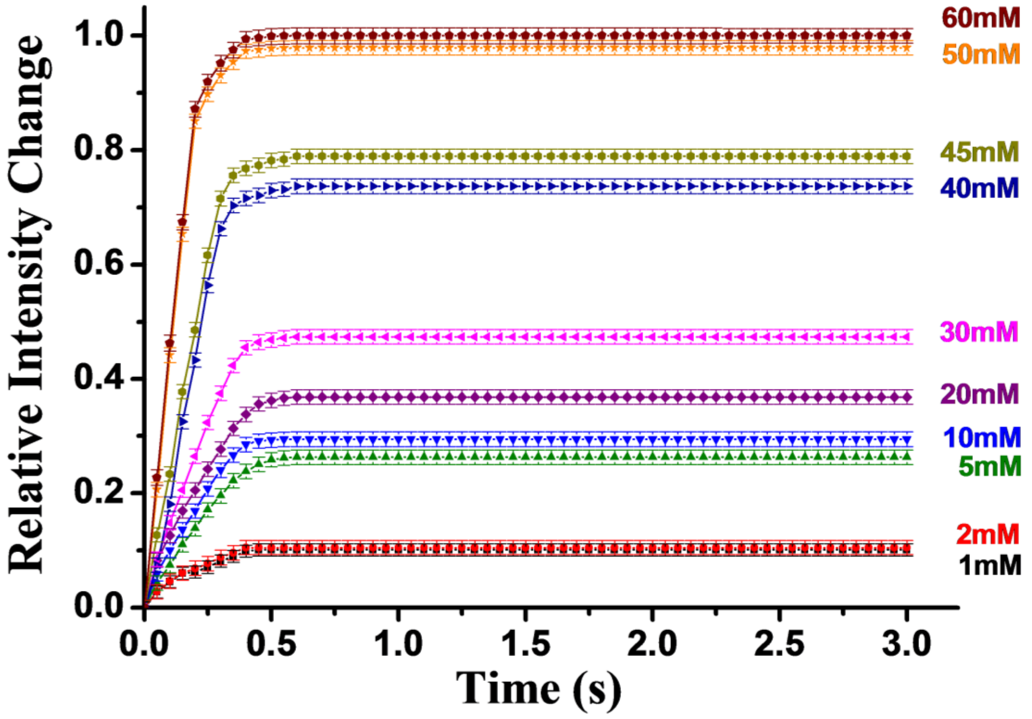

Figure S1 The kinetic behaviour of the glucose sensing system upon addition of different concentrations of glucose (N = 3).

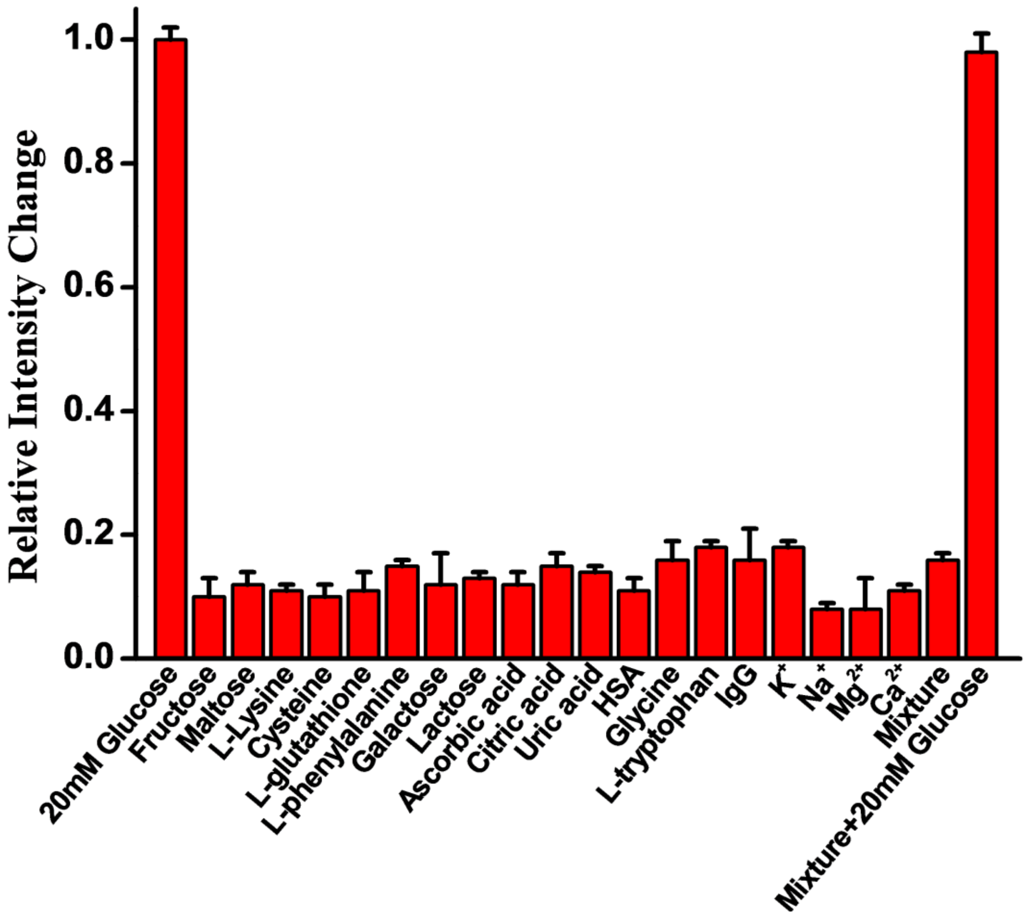

Figure S2 Selectivity analysis of the gel-encapsulated **B5** for detection of glucose. Evaluation of the selectivity for the glucose detection by using Fructose 310 μM, Maltose 120 mM, L-Lysine 3.7 mM, Cysteine 3 mM, L-glutathione 0.65 μM, L-phenylalanine 496.5 μM, Galactose 18 μM, Lactose 180 μM, Ascorbic acid 340 μM, Citric acid 540 μM, Uric acid 5 mM, HSA 0.075 mM, Glycine 4.9 μM, L-Tryptophan 78 μM, IgG 0.733 mM, K^+ 42.5 mM, Na^+ 1.43 M, Mg^{2+} 8.3 mM, and Ca^{2+} 25 mM. The error bar represents the standard deviation of three independent measurements. .

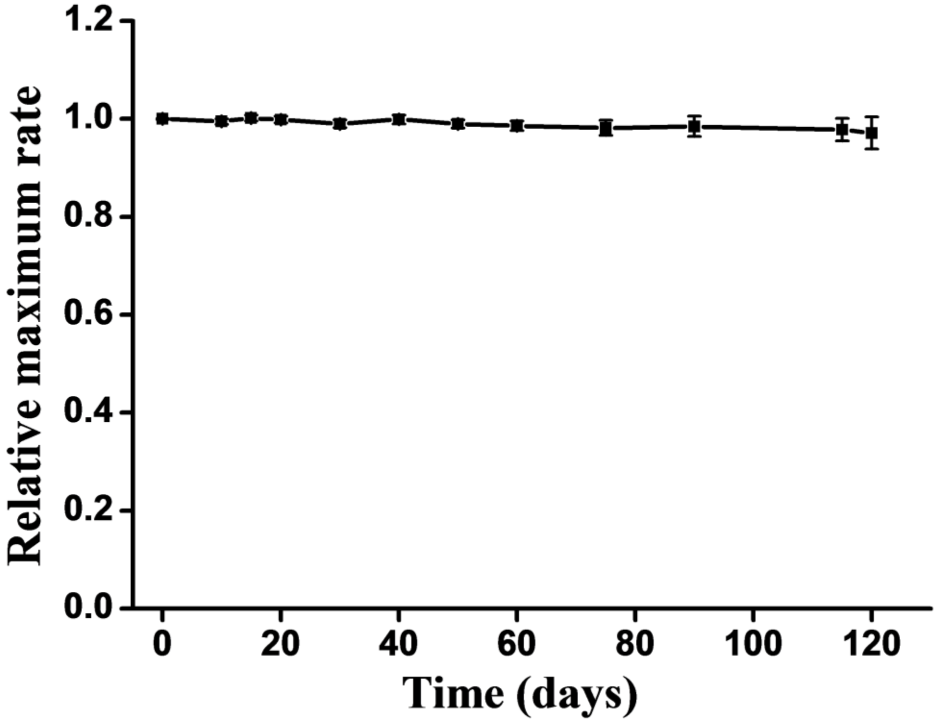

Figure S3 The storage stability of the gel-encapsulated **B5** for detection of 20 mM glucose during 120 days storage (N = 3). The biosensor was stored at 4 °C and then allowed to stand at room temperature until thawed prior to use.
